# P-2285. Factors Associated with Chagas Reactivation After Heart Transplantation: Retrospective Analysis at a Large Volume Academic Transplant Center

**DOI:** 10.1093/ofid/ofae631.2438

**Published:** 2025-01-29

**Authors:** Omer E Beaird, Ashrit Multani, Pryce Gaynor, Lauren Yanagimoto-Ogawa, Stephanie Fraschilla, Maya King, Jonathan G Smith, Ali Nsair, Margrit Carlson, Bernard M Kubak, Joanna M Schaenman

**Affiliations:** UCLA, Los Angeles, California; David Geffen School of Medicine at UCLA, Los Angeles, California; UCLA, Los Angeles, California; David Geffen School of Medicine at UCLA, Los Angeles, California; Ronald Reagan UCLA Medical Crnter, Los Angeles, California; UCLA Medical Center, Los Angeles, California; UCLA, Los Angeles, California; UCLA, Los Angeles, California; UCLA, Los Angeles, California; UCLA Med Cntr, LOS ANGELES, California; University of California Los Angeles, David Geffen School of Medicine, Los Angeles, California

## Abstract

**Background:**

Chagas cardiomyopathy (CC), caused by *Trypanosoma cruzi*, is a common indication for orthotopic heart transplantation (OHT) for patients born in Latin America, however post-transplant Chagas reactivation (CR) is common, occurring in ∼60% of cases. Serial *T. cruzi* PCR monitoring post-OHT with pre-emptive therapy is the current standard for managing post-transplant CR in part due to poor tolerability of benznidazole and nifurtimox. While this approach is often effective, poor accessibility can create delays in treatment, leading to adverse outcomes. We examined patients with CC who underwent OHT to evaluate factors associated with CR.

**Figure:**
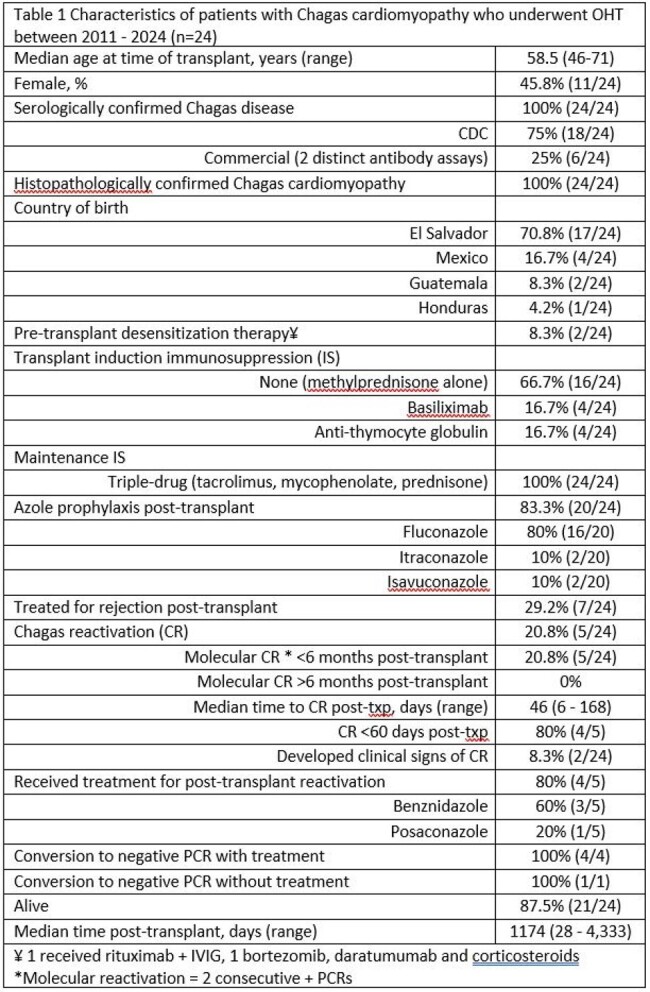

**Methods:**

We conducted a retrospective review of all patients with CC who underwent OHT at our center between January 2011 – May 2024, reviewing demographics, pre-transplant desensitization, induction and maintenance immunosuppression, rejection history, and presence or absence of azole prophylaxis.

**Figure:**
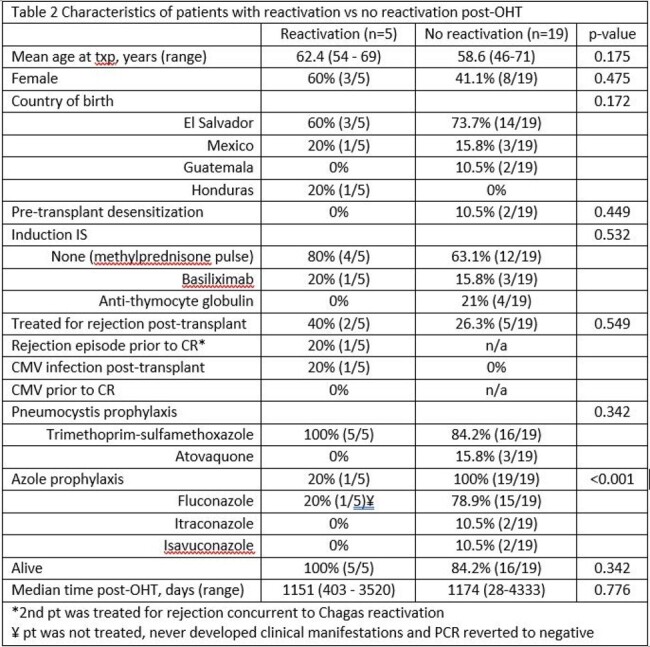

**Results:**

24 patients with confirmed CC were identified, whose characteristics are listed in Table 1. CR occurred in 5 (20.8%) patients, of whom 2 developed clinical manifestations. Asymptomatic CR occurred in 1/20 patients (5%) on azole prophylaxis, whereas 4/4 (100%) patients not on azole prophylaxis had CR (p< 0.001). Age, sex, induction immunosuppression, and treatment for rejection were not significantly associated with CR. Comparison of patients with and without CR is shown in Table 2.

**Conclusion:**

The rate of CR in our cohort was lower than expected based on published data. This may be related to the use of azole prophylaxis which we frequently use for prevention of coccidioidomycosis. Azoles have varying *in vitro* and *in vivo* antitrypanosomal activity. In randomized clinical trials (RCTs), posaconazole demonstrated ability to suppress molecular detection of *T. cruzi*. Our findings suggest that azole prophylaxis, including fluconazole, may have a protective effect against CR. Considering favorable cost and tolerability of fluconazole as compared to benznidazole and nifurtimox, these findings could have significant implications for post-transplant management of CC. Given the small number of patients with CR in our cohort, larger studies including RCTs are needed to validate this observation.

**Disclosures:**

Joanna M. Schaenman, MD, PhD, FAST, Eurofins Viracor: Honoraria|F2G: Grant/Research Support|MedCure: Advisor/Consultant|Moderna: Clinical trial support to institution|OneLegacy: Advisor/Consultant

